# The journey to recovery: unmet dental care needs in individuals with eating disorders

**DOI:** 10.1186/s40337-025-01376-x

**Published:** 2025-08-25

**Authors:** Ulrica Gidlund, Tove Hasselblad, Pernilla Larsson-Gran, Yvonne von Hausswolff-Juhlin, Nikolaos Christidis, Göran Dahllöf

**Affiliations:** 1 Department of Prosthetic Dentistry, Eastmaninstitutet, Public Dental Service, Folktandvården Stockholms Län AB, Dalagatn 11, 113 24 Stockholm, Sweden; 2 Department of Dental Medicine, Karolinska Institutet, Stockholm, Sweden; 3 Department of Prosthodontics, Faculty of Odontology, Malmö University, Malmö, Sweden; 4 Department of Clinical Neuroscience, Centre for Psychiatry Research, Karolinska Institutet, Stockholm, Sweden; 5 Stockholm Health Care Services, Region Stockholm, Stockholm, Sweden; 6 Center for Oral Health Services and Research, Mid-Norway, TkMidt, Trondheim, Norway

**Keywords:** Anorexia nervosa, Bulimia nervosa, Dental erosion, Eating disorder, Erosive tooth wear, Oral health, Reflexive thematic analysis

## Abstract

**Background:**

A well-established link exists between eating disorders and oral health issues such as dental erosion, caries, and tooth loss. However, little is known about how to best provide dental care for individuals with eating disorders. Therefore, current guidelines often suggest delaying dental treatment until after medical rehabilitation. This study aimed to explore the dental care experiences of patients with eating disorders, with the objective of finding ways to improve dental care and support for this patient group throughout the disease.

**Methods:**

Ten women (average age 36.7 years; SD ± 12.7; range 21–51), all recovered from an eating disorder (median duration of illness 12.5 years, range 4–25), participated in semi-structured interviews about their dental care experiences during and after their illness. Participants were purposefully sampled from a Swedish specialist dental clinic. Using inductive reflexive thematic analysis, the research team developed key themes to highlight important aspects of their narratives.

**Results:**

An overarching theme of unmet dental care needs marked the journey to recovery for the participants. Three major themes were constructed: (1) navigating uncharted territory, participants often felt isolated and lacked guidance from dental professionals on managing oral health during illness and recovery; (2) missed opportunities to build confidence and capability, dental care encounters frequently failed to address individual needs, with shame, cost, and limited support undermining confidence and self-efficacy; and (3) the importance of oral health in rebuilding hope and identity, restoring oral health was seen as vital to recovery, supporting self-esteem and a renewed sense of self.

**Conclusion:**

The study revealed ongoing unmet needs in dental care for the individuals examined. Participants’ experiences revealed uncertainty, missed opportunities for empowerment, and the vital role of oral health in restoring hope and identity. Oral health professionals should offer compassionate, precise, and personalized support, integrating oral health into the broader recovery process to enhance confidence and overall well-being for patients with eating disorders.

## Abstract (plain language summary)

Eating disorders are associated with poor oral health, particularly erosive tooth wear. Still, little is known about how people with eating disorders experience dental care at the beginning of their illness, during treatment, and after recovery. Therefore, dental treatment is often delayed until after medical rehabilitation, which may increase the risk of worsening oral health and lead to preventable suffering and high costs for both patients and healthcare systems.

This study aimed to fill these gaps of knowledge by interviewing women who had recovered from an eating disorder about their experiences with dental care. The ten participants shared that they faced considerable challenges in getting proper dental care during their illness and recovery. Many felt they were navigating unfamiliar territory without enough support or clear guidance from dental professionals. Common barriers included feelings of shame, financial struggles, and a lack of personalized treatment, which made dental visits stressful and discouraged them from seeking help.

Importantly, participants highlighted that restoring their oral health was vital for rebuilding self-esteem, and identity. Addressing these unmet dental care needs is essential for supporting both oral health and overall well-being throughout the journey to recovery.

## Background

Eating disorders (EDs) such as anorexia nervosa and bulimia nervosa affect over 200,000 people annually in Sweden, most often during adolescence and young adulthood [1]. EDs cause considerable psychological and physiological harm, leading to high disability and even death [2, 3].

Irreversible dental damage is a major, but often overlooked, consequence of EDs [4, 5]. Oral complications, including erosive tooth wear, caries, and tooth loss, are common due to nutritional deficiencies and behaviors like self-induced vomiting [6, 7]. Without timely intervention, these injuries not only persist but progressively worsen, leading to increased pain, eating difficulties, aesthetic concerns, and social withdrawal. This ongoing damage increases stigma, lowers quality of life, and incurs high costs for both patients and society [6–8].

Despite the high prevalence of severe oral health complications in patients with EDs [4–7], there is a notable lack of research and clinical guidelines, with only case reports published [9–13]. As a result, dental professionals often lack the evidence-based knowledge and practical tools necessary to provide effective, timely care for this vulnerable group [14]. Consequently, dental care is frequently delayed or deprioritized, and clinicians feel unprepared to address the complex needs of these patients [14, 15]. This leads to a passive approach, resulting in preventable oral suffering in addition to the ED.

Recent qualitative research from our group indicates that dental damage remains a visible, lingering scar after ED remission, which can deepen psychological distress and social inhibition [16]. By focusing on patient perspectives, this study aims to provide first-hand insights into the challenges, needs, and expectations of individuals with EDs in relation to dental care. The findings could help develop more effective, empathetic, and patient-centered dental care approaches for this group. Therefore, this study aimed to explore patients’ experiences with dental healthcare, guided by the research question: “How do dental patients with eating disorders perceive and interact with dental healthcare services during their illness and recovery, and after recovery?”.

## Methods

This study is part of a larger research project assessing oral rehabilitation in individuals with a history of eating disorders (ClinicalTrials.gov NCT06088847). As an initial step, we conducted a qualitative interview study to gain a deeper understanding of how patients with longstanding EDs engage with and experience oral health and dental care throughout their illness and recovery.

### Participants

Participants were recruited from patients referred for dental treatment at the Department of Prosthetic Dentistry, Eastmaninstitutet, Public Dental Service, Stockholm, between January 31, 2022, and January 18, 2023. Inclusion criteria included being 18 years or older, being in remission from an ED diagnosed by a psychiatrist according to DSM-5 [17] and having sufficient verbal proficiency to describe their experiences. Eligible patients were invited to participate after a dental examination at the clinic.

Sample size was determined based on the principles of information richness and methodological coherence described by Malterud et al. [18] and Braun and Clarke [19]. Before recruitment, a target range of 8–12 participants were set to ensure adequate depth and diversity.

Recruitment was challenging because some potential participants, even years after recovery, still found discussing their ED experiences emotionally triggering. Several expressed hesitation to engage in psychological conversations and preferred to focus on dental care rather than revisit psychological issues. This made recruiting participants difficult, despite their willingness to take part in clinical dental intervention studies. Ten interviews provided rich, nuanced data sufficient for a robust qualitative analysis, as supported by Braun and Clarke [19, 20]. The detailed narratives even allowed the division of the findings into two separate articles [16]. To prioritize participants’ well-being, we limited recruitment accordingly.

### Interview guide

An interview guide was developed through a pilot study, as described in Gidlund et al. [16], and used a semi-structured format. Interviews started with questions about participants’ oral health experiences related to their ED, then moved on to specific inquiries about dental care interactions, including their expectations, hopes, and concerns about their planned oral rehabilitation.

### Data collection

**Fig. 1:**
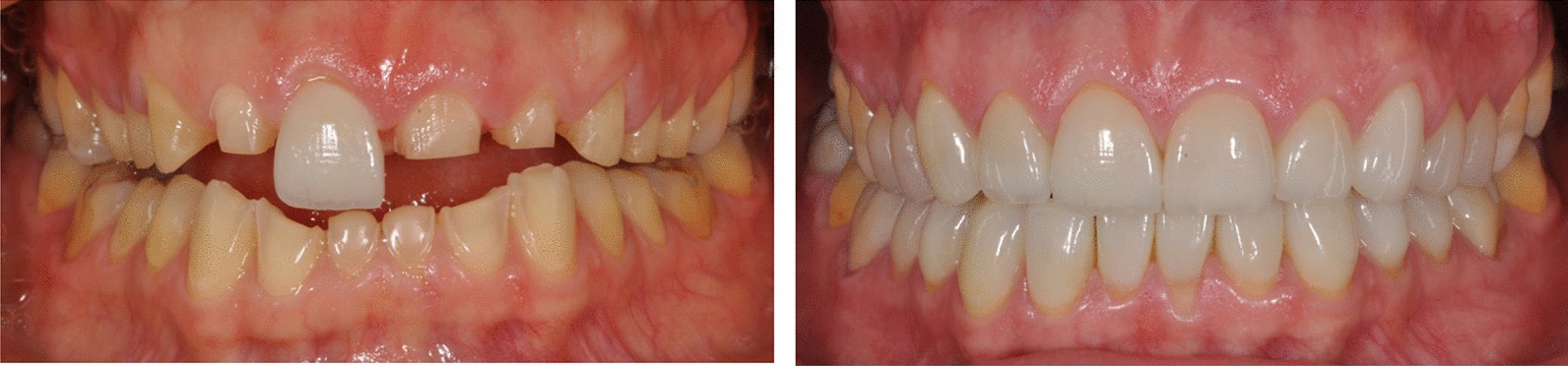
Permanent dentition showing severe erosive tooth wear and oral rehabilitation with porcelain crowns in a 40 year-old woman who has suffered from an eating disorder for 20 years. **A** permanent dentition showing preparation of teeth 15–25 and try-in of porcelain crown 11. **B** oral rehabilitation with porcelain crowns in teeth 15–25 and 35–45.

Ten female participants were enrolled in the study, aged 21–51 years (mean age 36.7 ± 12.7), with ED experiences spanning from 4 to 25 years (median 12.5) (Table 1). The participants had various dental issues, including missing teeth, dental caries, orofacial pain, impaired aesthetics, and severe erosive tooth wear (Fig. 1).

**Table 1:** Characteristics of the study participants

Sex	Age	Diagnosis	Duration of eating disorder (Years)	Secondary diagnoses	Teeth In Need Of Dental Restoration (*N*)
F	49	Bulimia Nervosa	25	Sober alcoholic	24
F	49	Bulimia Nervosa	25	ADHD	26
				Autism	
F	22	Bulimia Nervosa	6	–	14
F	21	Bulimia Nervosa	4	–	12
F	46	Bulimia Nervosa	20	Depression	6
F	32	Bulimia Nervosa	20	ADHD	21
				Bipolar disease	
				Autism	
F	23	Anorexia Nervosa	6	Rumination	23
				Reflux	
F	50	Bulimia Nervosa	15	–	22
F	24	Bulimia Nervosa	10	Bipolar disease	18
				PTSD	
				Autism	
F	51	Bulimia Nervosa	10	–	18

ADHD, attention deficit hyperactive disorder; PTSD, post-traumatic stress syndrome.

The interviews took place in a conference room at Eastmaninstitutet, separate from the dental clinic. A psychologist (TH) facilitated the interviews. TH allowed the participants to lead the conversation, intervening only with follow-up questions when necessary or when the discussion slowed. The interviews lasted from 13 to 81 min and were audio-recorded.

### Transcripts

All interviews were transcribed verbatim by a professional language service (Spoken, www.spokencompany.se). GD and UG verified the accuracy of the transcripts against the original audio recordings. To protect confidentiality, participants were assigned pseudonyms throughout the interviews, transcripts, and this publication. The original Swedish transcript codes were translated into English using CoPilot. Translation prompts were carefully designed to maintain strict fidelity to the source material, avoiding paraphrasing, summarizing, or interpretive changes. Alternative renderings were provided in parentheses where ambiguity existed. To ensure translation accuracy, all codes were back-translated into Swedish using the same model and prompts, demonstrating high fidelity in both meaning and tone, and resulting in consistent coding across both languages.

### Unit of analysis and rationale for reflexive thematic analysis

The unit of analysis in this study was the individual participant with a history of an ED who had experienced dental care during and after her illness. Each participant’s narrative, collected through semi-structured interviews, was treated as a whole to explore their unique experiences, challenges, and needs related to dental care. This person-centered approach enabled us to capture not only what occurred during dental visits but also how these experiences were perceived and understood by the individuals themselves, information often overlooked in quantitative or survey-based research.

To analyze the interview data, we used Braun and Clarke’s reflexive thematic analysis (TA) [21–27], a qualitative approach especially suited for exploring complex, subjective experiences. For dental professionals, this method offers useful insights into patients’ needs, expectations, and barriers to care, helping to develop more empathetic and effective clinical strategies for individuals with EDs.

### Reflexive thematic analysis

This study was grounded in a non-positivist, constructionist, and inductive paradigm, which influenced both the research question and the analytical strategy. Reflexive thematic analysis, as described by Braun and Clarke [19–27], was chosen for its strong alignment with this epistemological stance, enabling the development of patterns and themes directly from participant narratives.

After repeatedly reading the interview transcripts for familiarization, the research team, comprising a psychiatrist, a psychologist, and four dentists, collaboratively conducted initial coding. Codes were generated inductively from the data and were reviewed and refined through time-intensive group discussions. During the coding process, we consistently asked, “What is this an example of?” to ensure the codes captured meaningful patterns relevant to the research question.

In the later stages of analysis, themes were defined and named, following Braun and Clarke’s [23, 26] guidance, with analytic narratives created for each theme. These narratives conveyed the main story of each theme and how it contributed to the overall story of the dataset. This process helped ensure that themes were constructed from the coded data, consistent with the dataset as a whole, and addressed the research question [28, 29]. Our analytical procedures were reflexive and transparent, and we did not use consensus coding, inter-rater reliability measures, or a codebook, consistent with the principles of, and fidelity to, reflexive TA [27].

### Rigor and trustworthiness

All interviews were conducted either before or at the start of the participants’ oral rehabilitation. With two exceptions, participants had met the recruiting dentist (UG) only once before the study began. The dual role of UG as both clinician and researcher was explicitly addressed during data analysis to ensure the integrity of the analysis and to minimize undue influence on interpretation.

TH, a psychologist not involved in the participants’ clinical care, conducted interviews to encourage openness and minimize the influence of pre-existing clinical relationships.

Throughout the analysis process, the research team engaged in ongoing reflexivity, actively acknowledging and reflecting on our own clinical perspectives, social positions, and experiences [30]. We recognized that the researchers’ backgrounds and subjectivities inevitably influence the development of themes. For example, a dentist and a psychiatrist contributed extensive professional expertise in treating patients with EDs. These viewpoints were openly discussed and carefully examined, recognizing their potential to inform and shape our interpretations and the development of themes. Simultaneously, this expertise helped ensure relevance to both the research questions and the lived realities of the patient group. By maintaining transparency and reflexivity, we aimed to responsibly represent participants’ voices while acknowledging how our own researcher and clinical positions might influence the process.

We also recognize that participants’ choices to join the study might have been influenced by social desirability, which could have affected how they shared their experiences and the extent to which they reported the impact of unmet oral health needs. This approach to data collection and analysis aimed to balance the value of researcher expertise with a dedication to reflexivity and transparency, while acknowledging possible limitations in participant accounts.

### Ethics

Individual interviews were selected over focus groups to protect participant privacy and prevent unwanted disclosure of their histories with EDs and dental issues. This approach created a confidential and supportive environment for sharing sensitive experiences.

Participant selection focused on psychological safety, with thorough screening to reduce the risk of triggering ED relapse. Support protocols were developed in collaboration with Stockholm’s Center for Eating Disorders, ensuring access to help for any participant in distress. The research team conducted thorough monitoring and quickly reported adverse events to the study sponsor, allowing for prompt intervention if needed. These ethical measures highlight the research team’s dedication to participant safety and responsible research conduct.

## Results

**Fig. 2:**
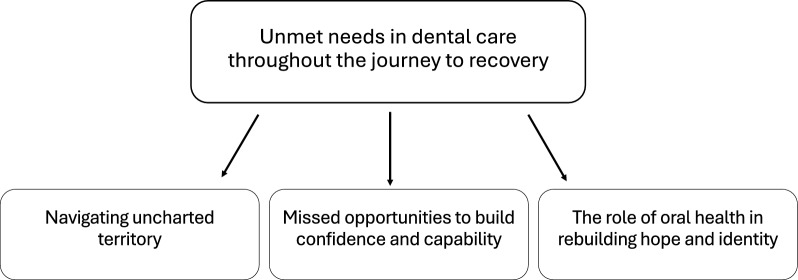
Thematic map showing the overarching theme and major themes developed from the data

The results of this study are based on interviews with young to middle-aged women who had experienced an eating disorder and poor oral health. All participants were referred to a specialist dental clinic and in need of extensive restorative treatment. The median number of teeth requiring crowns or composite resin restorations was 19.5, with a range of 6 to 26. Analysis of their narratives revealed an overarching theme: unmet needs in dental care throughout the recovery journey. This overarching theme was further described through three major themes: (1) navigating uncharted territory, reflecting the lack of guidance and personalized support; (2) missed opportunities to build confidence and capability, highlighting barriers such as shame, cost, and inadequate individualized care; and (3) the role of oral health in restoring hope and identity, emphasizing the importance of oral rehabilitation in participants’ recovery and self-esteem (Fig. 2).

### Unmet needs in dental care throughout the journey to recovery

The overarching theme of unmet needs in dental care throughout the recovery journey highlights the lack of individualized care experienced by individuals in this study at every stage, from illness onset to recovery. Despite frequent contact with dental services, participants reported that their specific needs related to their ED were often overlooked or insufficiently addressed. This left the participants feeling unsupported and vulnerable to ongoing oral health problems.

Several participants shared that receiving encouragement and information about potential specialist interventions could have helped them stay motivated during difficult times.

*… I was desperate… I mean, just getting this encouragement that you can get specialist help, and it’s possible to get [dental] crowns. That might have made me fight a little harder and see things more positively, rather than focusing on the negative aspects. It would have been encouraging. I think it is also important to be in a positive spiral to have the energy to reach goals; otherwise, you just end up like: Well, okay, now you’re almost at rock bottom.*

*# 7, 23 years*

While some younger participants reported positive experiences with empathetic oral health professionals (OHPs), nearly all described repeated consultations that were unhelpful and characterized by feelings of shame, condemnation, and blame.

*Everything just got worse and worse. It just escalated. And the shame got even greater, and I didn’t dare go to the dentist.*

*#10, 49 years*

For some, repeated negative experiences worsened a cycle of worry and hopelessness, eventually causing them to avoid dental care out of embarrassment and discomfort.

*My dental problems... did not really stop … it got a little worse each time [I visited the dentist]. Maybe I was ashamed … It was embarrassing to open my mouth. I haven’t felt particularly comfortable. Less comfortable every time****.***

*#7, 23 years*

#### Navigating unchartered territory

The theme navigating uncharted territory reflects participants’ experiences of entering dental care without guidance or support tailored to their needs as individuals with EDs. Many described feelings of uncertainty and isolation resulting from a lack of clear information from dental professionals about managing oral health during illness and recovery. Without personalized communication or structured care pathways, participants often felt unsure about what to expect or how their ED might influence dental treatment, which contributed to anxiety and reluctance to seek care.

Several participants reported feeling that dental professionals were themselves unsure or unprepared to address their specific concerns, which left participants feeling dismissed or unsupported.

*She [the dentist] just looked at me as if she had no idea how to handle the situation. She seemed even more scared than I was; she had no clue. So, it felt like I was sitting there, even crying, very sad and very worried, and they just said, “Yes, you have some damage, but there's nothing we can do. “They just sent me home with advice to stop vomiting and rinse my mouth. So, I’ve truly hated how I’ve been treated by dentists.*

*#4, 21 years*

Participants also emphasized the importance of greater understanding among dental professionals, especially regarding the emotional and psychosocial factors associated with EDs. They pointed out that empathy, rather than judgment, can help make the dental environment feel safer and more welcoming.

*… I think… it would be important to also have some education in the emotional part around these things [among OHPs]. Because if you feel that you might still dare to go there [to the dentist] and because it’s like exposing yourself in some way... then it will only be difficult if you are condemned … That would be a suggestion [to OHPs] … getting that knowledge. Because it is not just soft drinks and poor dental hygiene that cause dental erosion.*

*#10, 51 years*

Some individuals described a strong sense of perceived injustice, feeling their dental needs were not given equal consideration when damage was caused by an ED.

*… I think that if you are an addict … or have some other kind of physical illness, then you usually get help earlier... But as soon as it comes to this [EDs], it’s like you don’t get any help and I think it’s unfair.*

*#6, 32 years*

Participants often felt not only misunderstood but also perceived judgment or disapproval from dental professionals when discussing their oral health, which increased their vulnerability and anxiety. One participant mentioned that these reactions, combined with her own vulnerability, made her feel judged rather than supported.

*… I did not feel that there was truly any understanding of the problem that I have and that it became more like disapproval … it probably also has to do with my own anxiety, that I think it’s so hard too, so I probably feel very condemned.*

*#10, 51 years*

When professional guidance was lacking, many participants turned to online resources to better understand their oral health issues and possible treatments.

*Well, I started googling a bit about... erosion damage and such on the teeth… When I looked up my symptoms, the first thing it said was to be careful about your teeth, and that’s how I got into it… I googled and Google is huge!*

*#7, 23 years*

#### Missed opportunities to build confidence and capability

The theme of missed opportunities to build confidence and capability highlights how dental care encounters often failed to support or empower individuals with EDs in managing their oral health. Participants described how feelings of shame, financial barriers, and a lack of personalized support from dental professionals made dental visits difficult and often discouraging. Instead of receiving encouragement or practical guidance tailored to their needs, many felt their concerns were ignored or insufficiently addressed, which undermined their confidence and self-efficacy. This lack of targeted support not only limited their engagement with dental care but also led to ongoing uncertainty and avoidance.

*... I remember the first time I threw up, the first time I understood that now this had turned into binge eating, into a bulimia … I read and googled, and it said that the teeth can be destroyed, so I called my dentist and made an appointment, because I just needed to know if I had any injuries. The dentist did absolutely nothing. The only thing she did was prescribe mouthwash for me and said: Rinse your mouth after every time you vomit.*

*#4, 21 years*

Notably, this participant contacted her dentist, not her medical doctor, immediately after her first vomiting episode, but the support she received lacked the guidance needed to build self-efficacy. This initial contact was a key chance to build confidence and capability, yet, instead, meaningful support was missed, leaving her without the tools to manage her oral health effectively.

Many participants emphasized that early detection and support are crucial, highlighting that each missed opportunity for timely dental care leads to further oral damage, increased costs, and lost chances to empower patients in managing their oral health.

*More support is needed for those with eating disorders, especially regarding dental care. It is important to identify issues earlier and address the financial aspect. There is a need for increased assistance in this area because… it becomes very expensive. In the long run, it will cost more if people do not receive the right help early on.*

*#6, 32 years*

Most participants expressed frustration about the lack of clear explanations from dental professionals regarding the link between ED-symptoms and dental erosion. This absence of information contributed to feelings of guilt and shame and hindered their understanding of why behavioral changes were necessary, potentially delaying recovery.

*… I wish someone had done that earlier [explained the link between vomiting, stomach acid and dental erosion], but there was no dentist who did… it was just… you must stop, but you never understood why you had to stop, you only got guilt and shame heaped on yourself even more... Had it happened earlier, explanations and the consequences of it … I could have stopped vomiting or something, it could have been a contributing factor to getting well earlier.*

*#6, 32 years*

Our reflexive TA also uncovered participants’ desire for compassionate, patient-centered communication that recognizes their current situation rather than giving blunt directives. Many highlighted that the absence of empathy from OHPs created a gap in care, hindering effective and supportive connections that meet patients where they are in their recovery process.

*…I think more information to those who have this disease and explain to them… calmly and nicely, in a humble way, and not just like this: You must stop!*

*#6, 32 years*

Many participants described accessing dental care as confusing and exhausting, with unclear referral pathways and overwhelming bureaucracy contributing to feelings of frustration and helplessness. These systemic barriers hindered their ability to obtain necessary support and represented missed opportunities for dental care to empower and assist individuals managing ED-related oral health needs.

*… Everything feels incredibly bureaucratic and complicated…This constant uncertainty, searching, and struggling to find the right person is emotionally draining. It is a never-ending process of trying to figure out how to get what you need. I gave up countless times because I couldn’t understand what I was supposed to do… which has caused me a great deal of anxiety****.***

*#2, 49 years*

#### The role of oral health in rebuilding hope and identity

Participants described the restoration of oral health, now within reach, as a key factor in their recovery journey, with far-reaching effects on their self-esteem and sense of self. For many, oral rehabilitation was not just about physical repair but also a profound psychological turning point. Addressing visible dental damage helped reduce the lingering scar of the illness, enabling individuals to move beyond the stigma and shame linked to both their ED and its oral consequences. This theme emphasizes how dental care, when delivered with empathy, can support not only physical healing but also the restoration of self-esteem, dignity, and a renewed sense of belonging.

For many participants, restoring their oral health represented an important step toward reclaiming a part of themselves damaged by the ED. This recovery was closely connected to easing feelings of embarrassment and social stigma, reflecting a deeper psychological healing beyond just the physical aspect.

*… I just want to repair … I don’t have such high expectations because I just want to make sure that my teeth get well again so that they look ok, so that I don’t have to be embarrassed.*

*#6, 32 years*

Participants also expressed that regaining functional use of their teeth was a key goal, symbolizing a return to normalcy and improving quality of life, which is vital for rebuilding hope and identity after their illness.

*The only thing you want is to be able to use your teeth.*

*#2, 49 years*

All interviewees expressed a strong desire to no longer be preoccupied with their teeth or feel compelled to hide the damage caused by their illness. For many, achieving a sense of normality in their appearance reflected the internal progress of their recovery, allowing them to reclaim positive aspects of their identity beyond the visible signs of their ED.

*… to not have to think about it so much anymore and just be happy with my teeth and for them to look like when I was younger and when I hadn’t damaged them. Because you think about it daily, all the time, just because you see it all the time.*

*#3, 22 years*

Several individuals described oral rehabilitation as a milestone in their recovery journey, linking the process directly to a renewed sense of wholeness and closure. For many, addressing dental damage was not just a physical repair but also an emotional step toward overcoming distress and anxiety related to their ED.

*I think it’s … a big step to take [the planned oral rehabilitation], but at the same time, it feels like I must do it, because it’s like … part of becoming whole in some way, to deal with what is my worst, like apprehension, and fear and … to put an end to a chapter.*

*#10, 51 years*

## Discussion

This study aimed to explore how women with a history of EDs and poor oral health experienced dental care during and after their illness. Using in-depth interviews with ten women in remission from an ED, we developed themes that highlight ongoing knowledge gaps in dental care and areas for improvement. These findings indicate that, throughout the disease and even after medical recovery, participants encountered considerable challenges in accessing suitable dental support, navigating a system often lacking tailored guidance, empathy, and continuity. By emphasizing the voices of those with lived experience, this study provides valuable insights into how dental care practices and policies can be adapted to better meet the complex needs of patients with EDs throughout their recovery journey.

Participants described feeling isolated and unsupported while engaging with dental care during their illness and recovery. Consistent with previous research, the participants of this study were all required to wait until risk behaviors stopped before receiving restorative treatment, despite ongoing oral health deterioration [9–13]. This aligns with current Swedish guidelines, which suggest postponing dental treatment until after medical rehabilitation [1, 31]. Such delays can lead to worsening oral health, increased suffering, and higher costs for patients [5, 6, 32, 33].

As highlighted by systematic reviews and qualitative studies, OHPs themselves report uncertainty and difficulty in meeting the needs of this group, often due to a lack of clear, evidence-based guidelines and limited education on ED-specific dental care [7, 15, 34, 35]. Consequently, both patients and professionals are left “without a map or compass” to guide patients on their journey to recovery, as described in both Norwegian and UK contexts, emphasizing the need for structured, patient-centered guidance in dental care for individuals with EDs [36, 37]. Furthermore, the lived experience perspective described by Patterson-Norrie et al. [14] and Downs [38] emphasizes the importance of comprehensive, empathetic care that considers the whole patient, not just their ED.

Consistent with other research, many participants reported that dental encounters did not meet their individual needs, lacked tailored information, and faced financial barriers that reduced their confidence and willingness to seek care [15, 36, 37]. Stigma and shame were also major barriers, as reported by Gidlund et al. [16] and Wall et al. [39]. Hamilton et al. [40] identified that perceptions of healthcare professionals failing to recognize EDs as legitimate illnesses, alongside pervasive stigma, substantially impede individuals’ willingness to seek help*.*

Some participants believed that earlier, honest communication about the irreversible consequences of ED behaviors might have motivated them to change. In contrast, others felt such information would have been overwhelming or unhelpful during active illness. As shown in the study by Johansson et al. [36], many OHPs admitted difficulties discussing EDs with patients, fearing it could damage trust, and were often unaware of referral pathways to specialist ED care. This lack of support led participants to seek information from non-professional sources, sometimes resulting in harmful self-care practices, a finding consistent with Conviser et al. [41], who reported that less than a third of women with bulimia nervosa saw OHPs as their primary source of oral health information.

Recent studies by Tumba et al. [42] further support the importance of patient-centered, harm-reduction approaches, and Downs [43], who argue that care should focus on realistic, individualized goals and quality of life rather than a rigid expectation of full recovery, especially for those with longstanding EDs. These findings emphasize the need to develop evidence-based, empathetic approaches in dental care and to ensure empower patients, reduce stigma, and provide personalized support throughout the illness and recovery.

Restoring oral health was described as essential for regaining self-esteem, social confidence, and a sense of identity after years of illness. Phillipou [44] described hope as a protective factor, even stating that “hope must be the lantern that lights our path” in relation to the struggle of recovering from long-term anorexia nervosa. Participants valued normalization and the ability to move beyond preoccupation with their teeth, rather than striving for perfect aesthetics, a view also observed in studies of other patient groups with extensive dental treatment needs [45]. Supporting hope through compassionate care, clear information, and collaborative goal setting may reduce shame and empower patients to maintain their oral health, thereby improving both emotional well-being and resilience. Improved oral health could help participants regain parts of their identity, confidence, and social involvement that had been lost during years of illness, as described in qualitative studies and accounts of lived experience [14, 31, 37, 46].

### Study strengths and limitations

A key strength of this study is the successful recruitment and in-depth interviewing of ten women with a history of severe EDs and poor oral health, a group that is both vulnerable and often challenging to engage in research. The richness and nuance of the data were further enhanced by the involvement of a multidisciplinary research team, including specialists in dentistry, psychiatry, and psychology, which strengthened the credibility and depth of the analysis. The use of inductive reflexive TA, with careful attention to methodological rigor and conceptual coherence, enabled the development of meaningful themes from participants’ narratives.

However, the study has several limitations. The sample included only women in remission from an ED, all recruited from a single specialist clinic in Sweden, and all with experience of both EDs and poor oral health. Therefore, the findings may not accurately represent the views of individuals with diverse clinical backgrounds, those with active illnesses, or people from other treatment settings. Additionally, the study relied on participants’ self-reported experiences, which could have been influenced by memory challenges or a desire to present themselves in a favorable light. Nevertheless, using in-depth interviews aimed to promote honesty and openness in participants’ responses. Despite these limitations, the study provides valuable insights into the unmet dental care needs and challenges faced by individuals with EDs, and highlights areas for future research and clinical improvement.

## Conclusion

Our study uncovers ongoing unmet needs in dental care for individuals with eating disorders across all stages, onset, active phase, and recovery. Although participants often used dental services, these visits frequently lacked proper support, involved refusals of care, and offered no tailored guidance, leaving many to manage their oral health in isolation. Shame, stigma, economic challenges, and communication barriers further complicated their experiences, while missed chances to build confidence and capability undermined trust and engagement with oral health professionals. Participants emphasized that oral rehabilitation is a crucial component of recovery, helping to restore self-esteem and identity, and serving as a vital step toward achieving psychological well-being. These findings underscore the importance of empathetic, patient-centered, and proactive dental care throughout the illness, as well as the development of best practice guidelines that address the specific challenges faced by this patient group. More research is necessary to ensure dental care genuinely supports recovery and enhances the overall quality of life for individuals with eating disorders.

## Data Availability

For ethical reasons, the interview transcripts cannot be shared. The informed consent form signed by participants did not authorize publication or distribution of the transcripts.
